# Gastrodin Ameliorates Acute Rejection via IRE1*α*/TRAF2/NF-*κ*B in Rats Receiving Liver Allografts

**DOI:** 10.1155/2019/9276831

**Published:** 2019-11-20

**Authors:** Fangchao Yuan, Xuesong Xu, Yakun Wu, Shigang Duan, Hao Wu

**Affiliations:** ^1^Department of Hepatobiliary Surgery, The Second Affiliated Hospital of Chongqing Medical University, Chongqing 400016, China; ^2^Department of Hepatobiliary Surgery, Suining Central Hospital, Suining, Sichuan 629099, China; ^3^Department of Hepatobiliary Surgery, The Ninth People's Hospital of Chongqing, Chongqing 400799, China

## Abstract

**Background:**

Liver transplantation (LT) is currently an effective treatment for end-stage liver disease, but the occurrence of acute rejection (AR) is still the main problem to be solved. The present study aimed to evaluate the effect of gastrodin (GAS) on LT.

**Methods:**

Rat transplant models were established and divided into SHAM, LT, GAS-L (50 mg/kg GAS), and GAS-H (100 mg/kg GAS) groups. The liver function, inflammatory factors, liver histopathology, survival of rats, number of M2-type macrophages, liver cell apoptosis, and pathway proteins were assayed at 7 days and 14 days after the operations.

**Results:**

With increasing GAS concentrations, liver function, expression of proinflammatory factors in the liver, and expression of M2-type molecules in macrophages were significantly improved, and the survival time of rats was significantly prolonged (*P* < 0.05). All rats treated with low or high doses of GAS were judged to have nondeterministic acute rejection. Flow cytometry showed that liver cell apoptosis was decreased significantly in the GAS-L and GAS-H groups after GAS administration compared with apoptosis and differentiation in the LT group (*P* < 0.05). Expression levels of Caspase-3, Bad, and Bax proteins were decreased, and the expression of the antiapoptotic protein Bcl-2 was increased in the GAS-L and GAS-H groups (*P* < 0.05). Mechanistically, the ERS-related IRE1*α*/TRAF2/NF-*κ*B pathway was suppressed by GAS, and GAS acted mainly on intrahepatic macrophages to affect AR and reduce ROS production (*P* < 0.05).

**Conclusion:**

GAS ameliorated AR by inhibiting the IRE1*α*/TRAF2/NF-*κ*B pathway in LT.

## 1. Introduction

Liver transplantation (LT) is currently an effective treatment for end-stage liver disease, but the occurrence of acute and chronic rejection is still the dominant cause of poor quality of life for patients. Long-term administration of immunosuppressive agents in patients will inevitably lead to bacterial or viral infections, malignant tumors, and receptor survival failure [1, 2]. Therefore, inducing and maintaining long-lasting immune tolerance between grafts and recipients is still an effective way to solve this problem in the field of liver transplantation research. It is currently believed that the basis of acute rejection (AR) after transplantation is the cascade of inflammatory responses, and the occurrence of inflammatory reactions is closely related to the functional status of antigen-presenting cells [3].

Currently, there has been increasing awareness regarding the role of endoplasmic reticulum stress (ERS) in AR of LT. Inositol-requiring protein 1*α*- (IRE1*α*-) dependent signaling acts as a sensor of ERS and is crucial for controlling the most highly conserved UPR signaling pathway [4]. Under physiological conditions, IRE1*α* binds to GRP78 and is inactivated. In response to ERS, IRE1*α* dimerizes and phosphorylates itself following GRP78 dissociation, resulting in the activation of IRE1*α* and priming expression of a variety of ERS-regulated genes [5]. Recently, some evidence has demonstrated that silencing IRE1*α* could regulate the STAT1 and STAT6 pathways in macrophages to promote an M2 phenotypic shift and inhibit the activation of JNK and the downstream apoptotic events in NAFLD [6, 7]. These results indicate that IRE1*α* is a vital mediator of oxidative stress and apoptosis and may be a potential target to induce immune tolerance in LT.

**Figure 1:**
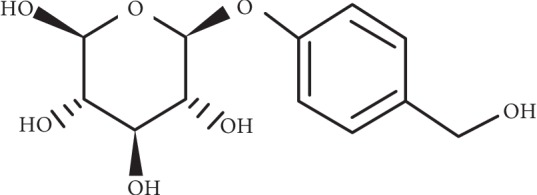
Chemical structure of GAS, whose molecular weight is 286.3 and molecular formula is C13H18O7.

In recent decades, numerous candidate treatments have been examined, including various herbal and natural products, which are considered safe and effective for improving quality of life with either no or few side effects [8]. Gastrodin (GAS), a natural phenol, is one of the major bioactive components extracted from the Chinese herb *Gastrodia elata* BI (Figure 1). Initially, GAS has been demonstrated to be a calcium channel blocker and can impede intracellular Ca^2+^ overload [9, 10]. In traditional medicine, GAS has been extensively used to treat cardiovascular and cerebrovascular diseases, such as stroke, dementia, and hypertension [11, 12]. Recent studies have indicated that GAS possesses comprehensive pharmacological functions, including antioxidative effects, anti-inflammatory effects, neuroprotective effects, and hypoxia tolerance [13]. GAS, as a potent antioxidant and free radical scavenger, can prevent uncoupled oxidative phosphorylation by activating the PTEN/PI3K/AKT and NF-*κ*B pathways to reduce cell apoptosis [14]. In liver diseases, GAS improved HCD-induced NAFLD by activating the PPAR*α* pathway or AMPK/Nrf2 pathway, thereby reducing liver oxidative stress and proinflammatory responses [15]. In addition, GAS is also implicated in anticancer immunomodulatory activity. It was indicated that GAS repressed transplanted H22 hepatic ascitic tumor cell growth by promoting NF-*κ*B-mediated gene transcription in CD4+ T cells [16]. Although GAS has been shown to have beneficial effects on the various ailments mentioned above, little is known about its function on immune tolerance in liver transplantation and the related signaling mechanisms. Therefore, we aimed to determine whether gastrodin can attenuate AR induced by allografts and to identify the molecular mechanisms that may be responsible for immune tolerance in LT.

## 2. Methods

### 2.1. Experimental Animals

Male Lewis donor rats (180–200 g) and male BN recipient rats (180–200 g) were purchased from the Experimental Animal Center of Chongqing Medical University (Chongqing, China). All mice were housed in cages in a room with a controlled temperature of 23°C and humidity of 60% under a 12 h light/12 h dark cycle. Mice had *ad libitum* access to food and water. Animal experiments were conducted in accordance with the guidelines of the China Association of Laboratory Animal Care.

### 2.2. Orthotopic Liver Transplant Models

An improved Kamada's two-cuff method with minor modifications was used to perform orthotopic liver transplantation as previously described [17]. Gastrodin (Cat. no. 62499-27-8; purity >99%) was purchased from Shanghai Winherb Medical Science Company (Shanghai, China). Experimental rats were randomly divided into the following 4 groups: SHAM group (*n* = 20) rats underwent laparotomy with exposure of the portal vein and injection of 2 ml of PBS via the caudal vein daily for 1 week after surgery; liver transplantation (LT) group (*n* = 20) rats underwent liver transplant operations and injection of 2 ml of PBS via the caudal vein daily for 1 week after surgery; low dose of gastrodin (GAS-L) group (*n* = 20) rats underwent liver transplant operations and injection of 2 ml of PBS plus 50 mg/kg GAS [18] via the caudal vein daily for 1 week after surgery; and high dose of gastrodin (GAS-H) group (*n* = 20) rats underwent liver transplant operations and injection of 2 ml of PBS plus 100 mg/kg GAS [18] via the caudal vein daily for 1 week after surgery. Rats in each group were euthanized at 7 d and 14 d after the operations. Blood and liver tissues were collected for analysis. The survival time in each group was recorded.

### 2.3. Cell Isolation and Purification

The livers of rats were perfused in situ with 10 ml PBS at 37°C for 3 min as previously described [19]. Then, liver tissues were dissolved in complete medium containing 0.5% type IV collagenase (Sigma-Aldrich; Merck KGaA, Darmstadt, Germany) at 37°C for 30 min. The liver homogenate was filtered through a 200 mesh sieve to remove undigested tissue, and the cell suspension was retained. The cell suspension was centrifuged at 300 × g for 5 min at 4°C. The supernatant was removed, and the precipitate was reserved. The precipitate was then resuspended in complete medium and centrifuged at 300 × g for 5 min at 4°C. The supernatant was removed, and the precipitate was reserved. The precipitate was then resuspended in complete medium and centrifuged at 300 × g for 5 min at 4°C. The supernatant was transferred into a new tube and centrifuged at 300 × g for 5 min at 4°C, the supernatant was removed, and the precipitate was reserved. The cell precipitates were cultured in complete medium for 4 h in a 5% CO_2_ atmosphere at 37°C. Then, the nonadherent cells were removed, and the adherent cells were intrahepatic macrophages. Intrahepatic macrophages were cultured in vitro, and then cells were collected for further analysis.

Dendritic cells (DCs) were then isolated using CD11c microbeads (>90% purity) and allowed to spontaneously mature overnight as previously described [20]. Then, the cells were collected for further analysis.

### 2.4. Detection of Liver Function and Inflammatory Factors

Blood samples were collected from recipient rats at the indicated time points following centrifugation at 2000 × g for 10 min at 4°C. Serum alanine aminotransferase (ALT) and aspartate aminotransferase (AST) levels were measured at 7 and 14 days after LT using a standard automatic biochemistry analyzer in the Clinical Biochemical Laboratory of Chongqing Medical University. IL-1*β*, MCP-1, TNF-*α*, and IL-10 levels were measured by ELISA according to the manufacturer's standard protocols (eBioscience, San Diego, CA). Absorbance was read on a Multiskan FC plate reader and analyzed with the SkanIt software for Multiskan FC (Thermo Fisher Scientific, Schwerte, Germany).

### 2.5. Liver Histopathology

Liver tissues were fixed in formalin, embedded in paraffin, and sectioned into 4 *μ*m thick sections. Sections were deparaffinized and dehydrated, and then hematoxylin staining was performed for 15 min. After rinsing with running water for seconds, sections were stained with eosin for 30 min. After rinsing with running water for seconds, sections were dehydrated with different concentrations of ethanol and xylene and sealed with neutral gum, and pathological changes were observed under the optical microscope. The Rejection Activity Index (RAI) scores (up to 9) are as follows: RAI<3 is classified as nondeterministic acute rejection, RAI = 3∼5 is classified as mild rejection, RAI = 5∼7 is classified as moderate rejection, and RAI = 7∼9 is classified as severe rejection reaction [21].

### 2.6. Immunofluorescence

Sections were deparaffinized and hydrated before antigen retrieval in 10 mM citric acid buffer. Then, sections were incubated in 1% Triton X-100 for 15 min. After eliminating endogenous peroxidase activity with 3% hydrogen peroxide for 15 min, sections were rinsed with PBS and incubated with primary anti-CD204 (cat. no. ab123946; 1 : 50; Abcam Inc.) and anti-CD206 (cat. no. ab125028; 1 : 50; Abcam Inc.) at 4°C overnight. After that, the sections were washed with PBS and incubated with specific secondary antibodies for 30 min at room temperature. Sections were then washed with PBS and sealed with Fluoromount-G™ Slide Mounting Medium (SouthernBiotech, Birmingham, AL, USA) as described before [22]. Images were taken using a fluorescent microscope and were analyzed using an Image Analysis system, version 11.0.

### 2.7. Flow Cytometry

Liver single-cell suspensions were harvested and washed twice with precooled PBS. The cell precipitate was then resuspended in binding buffer, and the cells were stained with annexin V and PI (cat. no. A026; GeneCopoeia Inc.) or fluorochrome-labeled CD204 (cat. no. ab123946; 1 : 50; Abcam Inc.), CD206 (cat. no. ab125028; 1 :50; Abcam Inc.), F4/80 (cat. no. ab6640; 1 : 50; Abcam Inc.), CD68 (cat. no. ab201340; 1 : 50; Abcam Inc.), MHC-II (cat. no. ab23990; 1 : 50; Abcam Inc.), and CD11c (cat. no. ab11029; 1 : 50; Abcam Inc.) followed by lucifugal incubation in the dark for 15 min at 37°C. Flow cytometric data were acquired using FACSCalibur and analyzed using the CellQuest version 5.1 software (BD Biosciences, Franklin Lakes, NJ, USA).

### 2.8. Western Blot Analysis

Cell or liver homogenate proteins of the above groups were extracted, and the concentrations were measured with BCA Protein Assay Kit (Sangon Biotech Co., Ltd., Shanghai, China). Samples were denatured for 10 min at a temperature of 100°C. A total of 40 *μ*g protein was loaded per lane, separated using 12% SDS-PAGE gel, and then electrotransferred onto polyvinylidene difluoride membranes. Membranes were blocked using 5% nonfat milk for 1 h and then incubated at 4°C overnight with primary antibodies against Bad (cat. no. ab32445; 1 : 1000; Abcam Inc.), Bax (cat. no. E63; 1 : 1000; Abcam Inc.), Caspase-3 (cat. no. ab2302; 1 : 1000; Abcam Inc.), Bcl2 (cat. no. ab196495; 1 :1000; Abcam Inc.), p-IRE1*α* (cat. no. ab48187; 1 : 1000; Abcam Inc.), IRE1*α* (cat. no. ab37117; 1 : 2000; Abcam Inc.), TRAF2 (cat. no. #4712; 1 : 1000; CST Inc.), p-I*κ*B*α* (cat. no. #2859; 1 : 1000; CST Inc.), I*κ*B*α* (cat. no. #9242; 1 : 1000; CST Inc.), p-p65 (cat. no. #3033; 1 : 1000; CST Inc.), p65 (cat. no. sc-71675; 1 : 2000; Santa Cruz Inc.), PPAR*γ* (cat. no. ab209350; 1 : 1000; Abcam Inc.), Arg1 (cat. no. ab60176; 1 :1000; Abcam Inc.), and iNOS (cat. no. ab15323; 1 : 2000; Abcam Inc.). The membranes were blotted with species-matched secondary antibodies. Protein bands were visualized using the BioRad ChemiDoc™ XRS system (Hercules, CA). All images were analyzed using the NIH ImageJ software.

### 2.9. Immunohistochemical Evaluation

Liver tissues were fixed in 4% paraformaldehyde, dehydrated, and subjected to heat-induced antigen retrieval using citrate. Then, the sections were incubated in 1% Triton X-100 for 15 min. After eliminating endogenous peroxidase activity with 3% hydrogen peroxide for 15 min, the sections were blocked with goat serum albumin at room temperature for 1 h. Then, the sections were incubated overnight at 4°C with p-IRE1*α* (cat. no. ab48187; 1 : 100; Abcam Inc.), TRAF2 (cat. no. #4712; 1 : 100; CST Inc.), and p-p65 (cat. no. #3033; 1 : 100; CST Inc.). The sections were washed and incubated with species-matched secondary antibodies (1 : 200 dilution) for 1 h at room temperature. The sections were washed with PBS, treated with 3,3′-diaminobenzidine for 5 min at room temperature, stained with hematoxylin for 30 sec at room temperature, and washed with flowing water for seconds. Following dehydration, sections were sealed with neutral resin, and specific staining was visualized by light microscopy as described before [22].

### 2.10. Measurement of Intracellular Reactive Oxygen Species (ROS) Levels

ROS production was assessed after staining the cells with CM-H2-DCFDA as described before [23]. After washing the cells with PBS, CM-H2-DCFDA (0.25 *μ*M) and PI (3 mg/ml) were added to each well. Cells were incubated for 10 min at 37°C, and then ROS levels and cell viability were simultaneously analyzed by flow cytometry.

### 2.11. Statistical Analysis

All values are expressed as mean ± SD. Data were analyzed using an unpaired two-tailed Student's *t*-test or one-way analysis of variance with a post hoc test. The SPSS 22.0 software was used in all statistical analyses. A *P* value less than 0.05 was required for results to be considered statistically significant.

## 3. Results

### 3.1. GAS Ameliorates the Inflammatory Response in Liver Transplantation and Improves Survival Time in Rats

**Figure 2:**
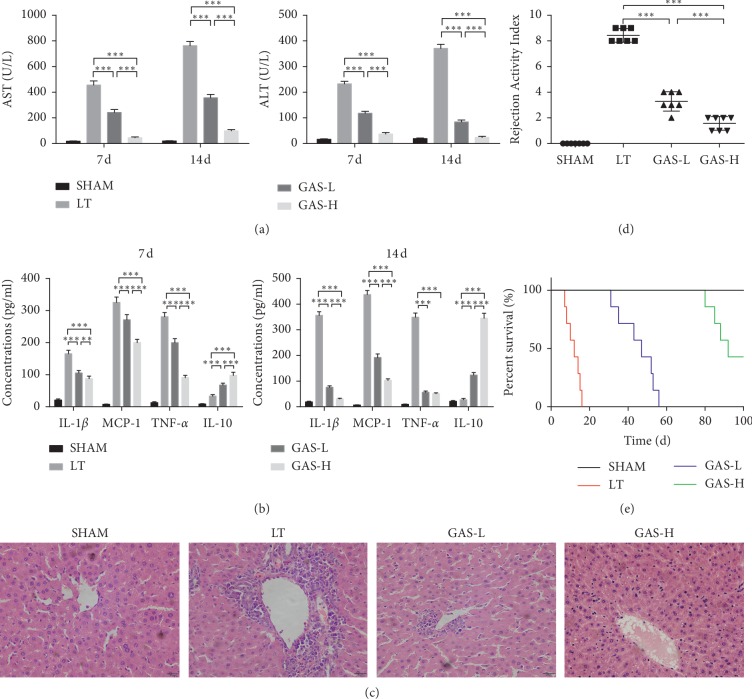
GAS ameliorates inflammatory response of liver transplantation and promotes survival time in rats. (a) Serum concentrations of ALT and AST in GAS treatment groups were significantly decreased after LT (*n* ≥ 5, ^*∗∗∗*^*P* < 0.001). (b) Inflammatory cytokines including IL-1*β*, MCP-1, TNF-*α*, and IL-10 were significantly decreased in GAS treatment groups (*n* ≥ 5, ^*∗∗*^*P* < 0.01, ^*∗∗∗*^*P* < 0.001). (c) Hematoxylin and eosin staining for observing the pathological damage. Visible focal necrosis and vacuolization of the liver parenchyma with lymphocyte infiltration were observed in the LT group, while GAS treatment groups had a normal architecture (magnification: ×400). (d) Banff schema for Rejection Activity Index (RAI) 14 d postoperatively. GAS treatment groups were judged as nondeterministic acute rejection (*n* ≥ 5, ^*∗∗∗*^*P* < 0.001). (e) Rat survival time was observed and analyzed using the log-rank test. Values represent mean ± SD of at least three independent experiments.

To test the effect of GAS on the acute rejection response in LT, the rats were treated with low or high doses of GAS after the operations. Our results showed that GAS protected transplant livers from being damaged by the acute rejection response. GAS improved liver function, decreased proinflammatory factor (IL-1*β*, MCP-1, and TNF-*α*) secretion, and promoted anti-inflammatory factor (IL-10) secretion in liver tissue homogenates 7 d and 14 d postoperatively (Figures 2(a) and 2(b)). Moreover, visible focal necrosis and vacuolization of the liver parenchyma with lymphocyte infiltration were observed in the LT group, but GAS improved the pathological changes in liver tissues with increasing concentrations (Figure 2(c)). According to the Banff schema, all rats treated with low or high doses of GAS were judged to have nondeterministic acute rejection, and the survival times of rats treated with GAS were markedly extended, especially in the GAS-H group (the survival time was over 100 d) (Figures 2(d) and 2(e)).

### 3.2. GAS Can Protect against Liver Cell Apoptosis and Promote M2-Type Polarization of Macrophages after Liver Transplantation

**Figure 3:**
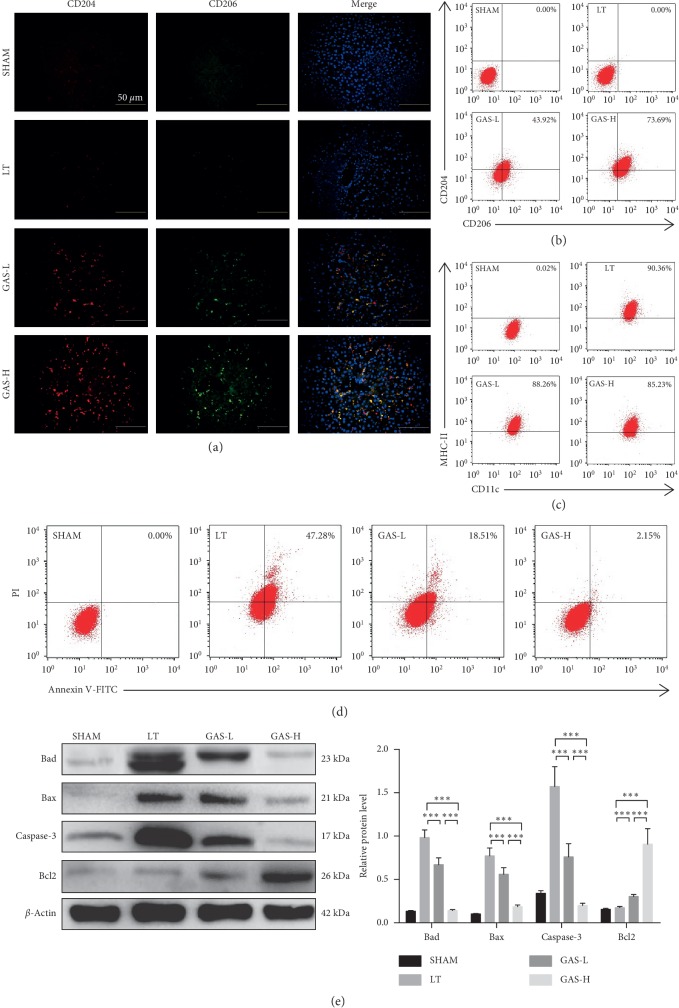
GAS protects against liver cell apoptosis and promotes M2-type polarization of macrophages after liver transplantation. (a) Immunofluorescence for observing CD204 (red) and CD206 (green) expressions in liver tissues 14 d postoperatively. More positively labeled M2-type macrophages were observed in GAS treatment groups (magnification: ×400, scale bars: 50 *μ*m). (b) The numbers of CD204(+)CD206(+) intrahepatic macrophages were increased in GAS treatment groups 14 d postoperatively with flow cytometric analysis. (c) Dendritic cells (DCs) were isolated from liver tissues 14 d postoperatively in each group. There was no significant difference in the numbers of CD11c(+)MHC-II(+) DCs between the LT group and the GAS group 14 d postoperatively with flow cytometric analysis. (d) Liver cell apoptosis in GAS treatment groups was decreased 14 d postoperatively using annexin V/PI staining by flow cytometric analysis. (e) Western blot was performed using liver tissues collected 14 d postoperatively. The expression of Bad, Bax, and Caspase-3 was decreased, and Bcl2 was increased in GAS treatment groups (*n* ≥ 5, ^*∗∗∗*^*P* < 0.001). Values represent mean ± SD of at least three independent experiments.

M2-type macrophages have weak antigen-presenting ability, express specific phenotypic molecules, such as CD204, CD206, and Arg1, and play an important role in immune tolerance [24]. We then determined whether GAS treatment can induce conversion of intrahepatic macrophages to M2-type macrophages. At 14 d after transplantation, the immunofluorescence in liver tissues showed that the expression of M2 phenotypic molecules (CD204 and CD206) in the LT group was extremely low, and with increasing GAS concentrations, the expression of CD204 and CD206 in liver tissues increased gradually (Figure 3(a)). The number of M2-type macrophages in the intrahepatic macrophage population determined by flow cytometry showed the same result as before (Figure 3(b)). To explore the effect of GAS on DCs' antigen-presenting ability, we isolated DCs from each group. CD11c was known to be a specific phenotype of DCs, and flow cytometry confirmed that there was no significant difference in the numbers of CD11c(+)MHC-II(+) DCs between the LT group and the GAS group (Figure 3(c)). Annexin V/PI staining indicated that the AR-induced hepatic apoptosis rate was decreased in the GAS groups compared to that in the LT group without GAS treatment (Figure 3(d)). Additionally, with increasing concentrations of GAS, the expression of proapoptotic proteins (Bad, Bax, and Caspase-3) was conspicuously suppressed, and the expression of antiapoptotic protein (Bcl2) was elevated in GAS-L and GAS-H groups (Figure 3(e)).

### 3.3. GAS Can Alleviate Acute Rejection Injury via Inhibiting the IRE1*α*/TRAF2/NF-*κ*B Pathway

**Figure 4:**
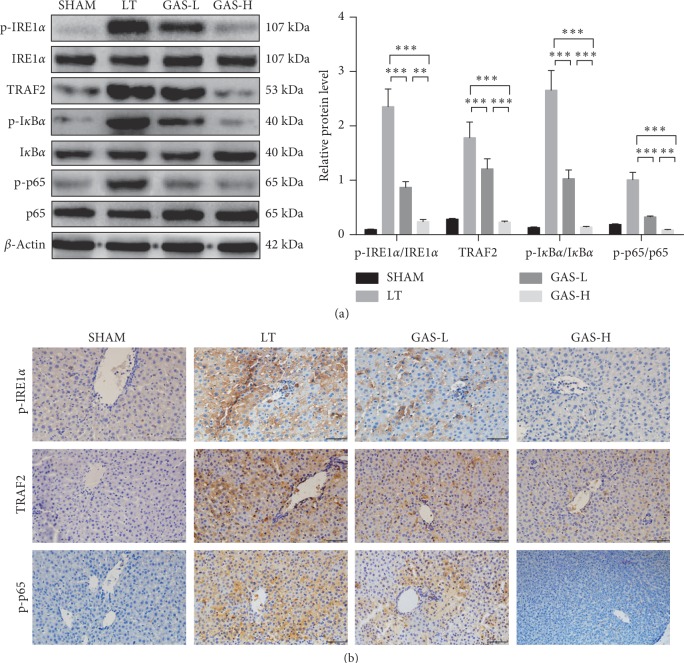
GAS alleviates acute rejection injury by inhibiting the IRE1*α*/TRAF2/NF-*κ*B pathway. (a) Western blot was performed using liver tissues collected 14 d postoperatively. The expression of p-IRE1*α*, TRAF2, p-I*κ*B*α*, and p-p65 proteins was decreased in GAS treatment groups (*n* ≥ 5, ^*∗∗*^*P* < 0.01, ^*∗∗∗*^*P* < 0.001). (b) Immunohistochemistry for observing the expression of p-IRE1*α*, TRAF2, and p-p65 in liver tissues 14 d postoperatively, which was significantly decreased in GAS treatment groups (magnification: ×400). Values represent mean ± SD of at least three independent experiments.

ERS is a self-protective body response, but extensive ERS reaction can cause pathological changes in the intracellular environment [25]. IRE1 is an important component in the activation of unfolded proteins during ERS. It was indicated that IREl*α* activates the NF-*κ*B pathway and JNK pathway by binding to TRAF2 to induce the inflammatory response and apoptosis [26]. To examine whether GAS could inhibit the ERS-related IRE1*α*/TRAF2/NF-*κ*B pathway in LT, we evaluated ERS pathway proteins in liver tissues at 14 d after transplantation. The expression levels of p-IRE1*α*, TRAF2, p-I*κ*B*α*, and p-p65 proteins were significantly increased in the LT group, whereas GAS treatment suppressed the expression of the aforementioned proteins (Figure 4(a)). The same results were determined with immunohistochemistry (Figure 4(b)).

### 3.4. GAS Acts Mainly on Intrahepatic Macrophages in Liver Transplantation

**Figure 5:**
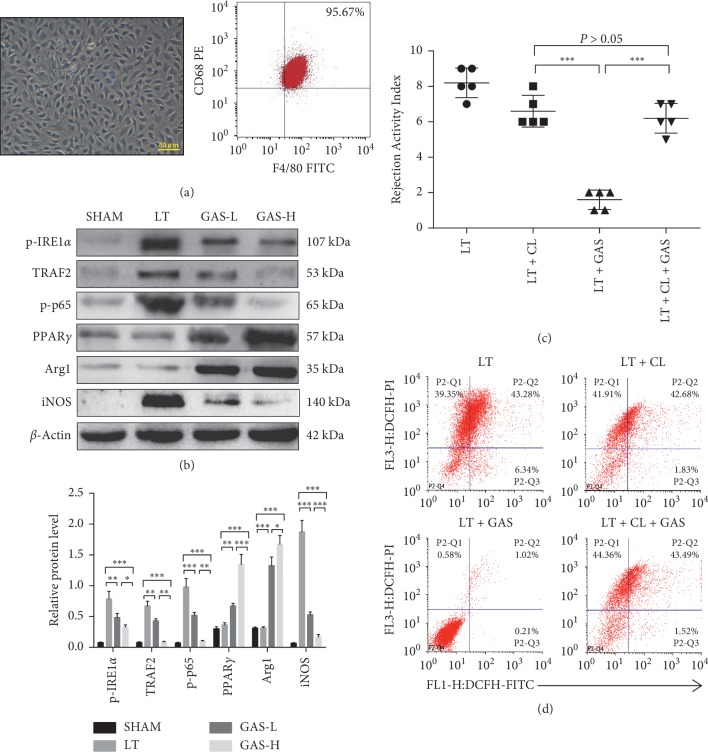
GAS acts mainly on intrahepatic macrophages in liver transplantation. (a) Intrahepatic macrophages were isolated from liver tissues 14 d postoperatively in each group. Intrahepatic macrophages mostly showed long spindle-shaped growth after cell attachment (magnification: ×400, scale bars: 50 *μ*m), and the purity of macrophages was over 95% with flow cytometric analysis. (b) Western blot was performed using intrahepatic macrophages collected 14 d postoperatively. The expression of p-IRE1*α*, TRAF2, p-p65, and iNOS proteins was decreased, and the expression of PPAR*γ* and Arg1 was increased (*n* ≥ 5, ^*∗*^*P* < 0.05, ^*∗∗*^*P* < 0.01, ^*∗∗∗*^*P* < 0.001). (c) The LT group underwent liver transplantation surgery. LT + GAS group recipient rats were injected with 100 mg/kg GAS via the caudal vein daily for 1 week after surgery. LT + CL group donor rats were injected with clodronate liposomes (CLs; 10 mg/kg) via the caudal vein to destroy intrahepatic macrophages 24 h prior to surgery. LT + CL + GAS group donor rats were injected with clodronate liposomes (CLs; 10 mg/kg) via the caudal vein to destroy intrahepatic macrophages 24 h prior to surgery, and recipient rats were injected with 100 mg/kg GAS via the caudal vein daily for 1 week after surgery. All rats underwent analysis to determine the Rejection Activity Index (RAI) score 14 d postoperatively (*n* ≥ 5, ^*∗∗∗*^*P* < 0.001). (d) The groups were the same as described in (c). The flow cytometric analysis of ROS production in liver cells was done using CM-H2-DCFDA and PI double staining. Values represent mean ± SD of at least three independent experiments.

Intrahepatic macrophages are the largest group of antigen-presenting cells in vivo [22]. Therefore, the immune balance of intrahepatic macrophages is crucial for the microenvironment in LT. In the subsequent experiments, we isolated intrahepatic macrophages from liver tissues, and the purity of macrophages was over 95% (Figure 5(a)). Then, we determined related pathway proteins in each group. The results showed that the expression levels of p-IRE1*α*, TRAF2, p-I*κ*B*α*, and p-p65 were remarkably inhibited in the same way as described previously (Figure 5(a)). Furthermore, with increasing GAS concentration, the expression of PPAR*γ* and M2-type protein Arg1 in macrophages was enhanced, but M1-type protein iNOS in macrophages was decreased compared with that in the LT group (Figure 5(b)).

Clodronate liposomes (CLs; 10 mg/kg) stimulate the depletion of macrophages in the liver [27]. To determine whether GAS acts mainly on intrahepatic macrophages in vivo, donor rats were treated with CLs to destroy intrahepatic macrophages. The RAI score of liver grafts treated with CLs plus GAS was associated with moderate AR, and there were no significant differences in RAI scores between the CL group and the CL + GAS group, but the RAI score of liver grafts in the GAS group without CL pretreatment was identified as nondeterministic acute rejection, suggesting that depletion of macrophages cannot improve AR and that GAS acts predominantly on intrahepatic macrophages instead of other intrahepatic cells to affect AR in LT (Figure 5(c)). In addition, depletion of macrophages with or without GAS treatment in the liver was unable to decrease ROS production compared with that in the single LT group, and there were no significant differences in ROS production between the CL group and the CL + GAS group (Figure 5(d)). However, the GAS group without CL pretreatment showed overtly reduced ROS production in liver tissues (Figure 5(d)).

## 4. Discussion

The impairment of grafts and the release of proinflammatory cytokines/chemokines (especially IFN-*γ*) induce major histocompatibility complex II (MHC-II) expressed on almost all cells' surface. High expression of MHC-II on the surface of antigen-presenting cells presents antigen to T cells, promoting the development of adaptive immune responses [22]. The vast majority of immunosuppressive agents are currently available in the clinic, including neurocalcin inhibitors (CsA or FK506), cytotoxic drugs (mycophenolate mofetil), and corticosteroids, which are effective in controlling AR by inhibiting the activation and proliferation of CD4+ and CD8+ T cells rather than preventing chronic rejection and cancer progression in patients [28]. It was demonstrated that intrahepatic antigen-presenting cells (Kupffer cells and dendritic cells) are activated by various routes after LT, and activated antigen-presenting cells have high expression of MHC-II, M1 costimulatory molecules, and strong antigen-presenting ability while secreting Th1 cytokines that activate antigen-specific T cells to induce AR [29]. During the inflammatory stage, a wide range of inflammatory factors contribute to the apoptosis of liver cells, while the accumulation of apoptotic cells brings about “secondary necrosis” with release of toxic intracellular substances (such as nucleosome fragments, DNA, and histones). The accumulation of these substances activates macrophages to release inflammatory factors (such as TNF-*α*, IL-1*β*, and IFN-*γ*) and oxidative stress products, which exacerbate AR [30, 31]. Therefore, the prevention of AR after LT is beneficial for improving the survival rate of allografts [32, 33]. Pharmaceutical research has found that GAS, extracted from *Gastrodia elata*, has various biological activities, such as scavenging oxygen free radicals, regulating immunity, preventing platelet aggregation, and protecting cell membranes [11–13]. In cardiac ischemia-reperfusion mice, it was found that GAS could regulate miR-21 and the PI3K/AKT pathway and inhibit the downstream NF-*κ*B pathway to improve myocardial ischemia-reperfusion injury [34]. Moreover, GAS improves the apoptosis of human retinal endothelial cells caused by high glucose via regulating the SIRT1/TLR4/NF-*κ*B pathway [35]. However, the specific role of GAS in LT is still unclear. In this experiment, GAS was used to investigate its effects and related mechanisms in LT.

In the current study, we administered low and high doses of GAS into donor rats before surgery to observe the liver immune status after transplantation. The liver functions were significantly improved, the expression of the proinflammatory factors IL-1*β*, MCP-1, and TNF-*α* was decreased, and the expression of the anti-inflammatory factor IL-10 was gradually increased in rats treated with GAS after surgery. Moreover, GAS significantly improved the pathological changes of liver tissues and prolonged the survival rate of rats. Antigen-presenting cells undergo polarization of different properties in different microenvironments, turning into a subpopulation of cells with different molecular phenotypes and distinct functions. The polarization state of antigen-presenting cells directly affects the formation of the immune microenvironment in LT. It is currently believed that M1 and M2 macrophages are the two extremes of continuous differentiation of mononuclear cells [36]: the M1 phenotype macrophage secretes proinflammatory factors and is involved in the progression of AR, while the M2 phenotype macrophage has a weak antigen-presenting ability and secretes anti-inflammatory factors, such as IL-10, which is involved in the formation of immune tolerance. The present study assessed that GAS could not apparently suppress the capability of antigen presentation in DCs after transplantation, whereas it promoted M2-type polarization of macrophages and protected against hepatocyte apoptosis in the liver, facilitating the formation of an immunotolerant microenvironment in LT.

IRE1*α*, a type I transmembrane protein, is an important component of the conserved signaling pathway involved in the activation of unfolded proteins during ERS. IRE1*α* is phosphorylated by associating with TRAF2, resulting in activation of the NF-*κ*B and JNK pathways. Activated JNK enters the nucleus, increases the expression of FasL and TNF, and initiates death receptor pathway-mediated apoptosis [5, 37]; NF-*κ*B is a nuclear transcription factor that is widely involved in important pathophysiological processes, such as the cellular inflammatory response, transformation, and apoptosis. Activation of NF-*κ*B is an essential step in the production of inflammatory factors by antigen-presenting cells [38]. A recent study found that administration of GAS inhibited ERS and reduced NLRP3 inflammatory bodies, improving cognitive dysfunction and depressive behavior in mice with diabetic encephalopathy [39]. Therefore, we examined the ERS-related IRE1*α*/TRAF2/NF-*κ*B signaling pathway. Our data showed that GAS injection reduced the expression of IRE1*α*, TRAF2, p-I*κ*B*α*, p-p65, Bax, Bad, and Caspase-3 proteins after transplantation and that the expression of the Bcl2 protein was elevated. These results were confirmed by immunohistochemistry analysis. The results demonstrated that GAS can alleviate AR by inhibiting the ERS-related IRE1*α*/TRAF2/NF-*κ*B pathway.

The immune balance of intrahepatic macrophages is vital for the formation of the microenvironment in LT. Thus, we isolated intrahepatic macrophages from liver tissues and demonstrated that GAS suppressed the IRE1*α*/TRAF2/NF-*κ*B pathway in intrahepatic macrophages and promoted M2-type polarization. To explore whether GAS acts on intrahepatic macrophages, we used CLs to destroy intrahepatic macrophages and found that depletion of macrophages cannot improve AR, suggesting that GAS acts mainly on intrahepatic macrophages instead of other intrahepatic cells to affect the AR in LT. ROS as a product of ERS comprise oxygen free radicals, such as superoxide, hydroxyl radicals, and peroxyl radicals. Excessive levels of ROS can cause an amplified cascade of inflammation and impairment of cell function [40]. LT has been shown to cause the production of free radicals after reoxygenation of the liver, leading to lipid peroxidation, hepatocellular necrosis, and graft nonfunction [41]. We found that depletion of macrophages with or without GAS treatment was unable to decrease ROS production in the liver, whereas the GAS group without CL pretreatment had overtly reduced ROS production in liver tissues, which indicated that GAS can improve the oxidative product secretion of macrophages and the intrahepatic macrophage-mediated hepatic oxidative stress response in LT.

In recent years, organ demand exceeds supply by tens of thousands. LT is considered the only option for end-stage liver disease, whereas many patients still face death because of the shortage of liver donor sources [42]. The application of immunosuppressive agents has unexpectedly adverse effects on patients with liver allografts. The present study verified that GAS can reduce inflammatory response after LT by inhibiting the IRE1*α*/TRAF2/NF-*κ*B pathway, promote M2-type polarization of intrahepatic macrophages to immune tolerance, protect the transplant liver from AR damage, and improve the survival time after transplantation in rats. The main findings of this study provided direct experimental evidence that GAS treatment could be a new approach to induce and maintain long-lasting immune tolerance in patients who have suffered from LT surgery.

## Data Availability

The datasets used and/or analyzed during the current study are available from the corresponding author on reasonable request.

## Abbreviations

AR: Acute rejection
Arg1: Arginase 1
ALT: Alanine aminotransferase
AST: Aspartate aminotransferase
CL: Clodronate liposome
DAB: Diaminobenzidine
DAPI: 4′,6-Diamidino-2-phenylindole
ERS: Endoplasmic reticulum stress
DCs: Dendritic cells
GAS: Gastrodin
GRP78: Glucose-regulated protein 78
JNK: c-Jun NH2-terminal kinase
IL-1*β*: Interleukin-1*β*
IL-10: Interleukin-10
IRE1*α*: Inositol-requiring protein 1*α*
iNOS: Inducible nitric oxide synthase
LT: Liver transplantation
MCP-1: Monocyte chemotactic protein 1
MHC-II: Major histocompatibility complex II
NAFLD: Nonalcoholic fatty liver disease
NF-*κ*B: Nuclear factor *κ*B
PPAR*γ*: Peroxisome proliferator-activated receptor *γ*
RAI: Rejection Activity Index
ROS: Reactive oxygen species
TNF-*α*: Tumor necrosis factor-*α*
TRAF2: TNF receptor-associated factor 2
UPR: Unfold protein response.
